# A Detailed Analysis of the Factors Influencing Neonatal TSH: Results From a 6-Year Congenital Hypothyroidism Screening Program

**DOI:** 10.3389/fendo.2020.00456

**Published:** 2020-07-17

**Authors:** Giulia Di Dalmazi, Maria Assunta Carlucci, Daniela Semeraro, Cesidio Giuliani, Giorgio Napolitano, Patrizio Caturegli, Ines Bucci

**Affiliations:** ^1^Section of Endocrinology, Department of Medicine and Aging Science, Center for Advanced Studies and Technology (CAST), G. D'Annunzio University, Chieti-Pescara, Italy; ^2^Department of Medicine and Aging Science, Center for Advanced Studies and Technology (CAST) and Translational Medicine, University of Chieti G. D'Annunzio, Chieti, Italy; ^3^Division of Immunology, Department of Pathology, The Johns Hopkins School of Medicine, Baltimore, MD, United States

**Keywords:** newborn screening, congenital hypothyroidism, thyroid stimulating hormone (TSH), thyroid diseases, preterm

## Abstract

**Background:** Neonatal thyrotropin (TSH) on dried blood spot (DBS), the most common screening strategy for primary congenital hypothyroidism (CH), is influenced by numerous factors that may hinder a true CH diagnosis. A second test can thus be performed to clarify the initial findings, although its application varies among screening programs.

**Objectives:** The aim of this study was to evaluate the effect of maternal and neonatal factors on neonatal TSH levels and offer practical screening recommendations.

**Methods:** We retrospectively analyzed screening data of 62,132 neonates born in Abruzzo, an Italian region considered mildly iodine deficient, between 2011 and 2016. We then performed a multiple linear regression to model the relationship between TSH (the dependent variable) and 13 independent variables extracted from blood collection cards.

**Results:** Most neonates (53,551 of 62,132, 86%) had normal TSH and no clinical indications for a second screening. A minority (1,423, 2.3%) had elevated TSH in the initial DBS, which was confirmed in 97 cases (7%) on a second screen. The remaining neonates (6,594, 10.6%) had a normal initial TSH but underwent a second test in accordance with screening protocols, and were found to have delayed TSH elevation in 23 cases (0.4%). Those 120 newborns (97 + 23), considered highly suspicious for primary CH, were referred to a pediatrician for confirmatory testing and excluded from subsequent analysis of factors influencing TSH levels. Sex (β regression coefficient, β = 1.11 female to male, 95% CI 1.09, 1.12) and age at collection (β = 0.78 day 5 to days 2–3, 95% CI 0.74, 0.83) affected neonatal TSH, suggesting the utility of specific nomograms. In addition, prematurity (β = 0.85 term to preterm, 95% CI 0.80, 0.91), dopamine use (β = 0.71, 95% CI 0.62, 0.81), and birth weight (β = 1.40 normal vs. very low, 95% CI 1.05, 1.89) strongly influenced neonatal TSH.

**Conclusions:** Neonatal TSH is influenced by several factors supporting the delineation of local sex- and age-adjusted TSH cutoffs, and the universal adoption of a second TSH test in neonates at risk of missed primary CH diagnosis.

## Introduction

Congenital hypothyroidism (CH) indicates the deficiency of thyroid hormones at birth, a deficiency that if unrecognized and untreated leads to severe intellectual disability and growth retardation. To recognize and promptly treat CH, a neonatal screening program was universally introduced, in Italy starting in 1977 and reaching full coverage in the early 1990s (1). The Italian CH screening is performed, as of December 31, 2016, by 26 regional and inter-regional clinical laboratories that screen newborns using either Dried Blood Spot (DBS) TSH (20 of 26, 77%), or both DBS TSH and total thyroxine (6 of 26, 23%) (2). Screening data are then reported to the Italian National Registry of Infants with Congenital Hypothyroidism, an office established by the Italian Ministry of Health in 1987.

The threshold value to determine whether TSH is elevated (hence the neonate at risk of primary CH or not) varies across Italian screening centers, with a range comprised between 6 and 12 mU/L (2). This threshold value has decreased over the years, resulting in a greater detection of primary CH cases, mostly mild (3).

Even when the initial TSH value is normal, recent guidelines (4) suggest collecting a second DBS specimen if the neonate belongs to categories known to delay the TSH elevation so as not to miss possible primary CH diagnoses. These categories include prematurity (i.e., gestational age <37 weeks), low and very low birthweight, twin delivery, congenital malformations, and chromosomal abnormalities such as down syndrome, blood transfusions, dopamine, total parenteral nutrition, or other conditions requiring admission to the neonatal intensive care unit (4, 5). Despite the guidelines, however, a lack of uniformity in screening programs for preterm, low, and very low birthweight neonates does occur, as recently reviewed in (6). Most authors advocate a second screening strategy, while others suggest either lowering the screening cutoff, using gestational age-adjusted cutoff, or testing for both TSH and T4 (6). It also remains unclear whether other factors such as sex, season of birth, and maternal history of autoimmune thyroid diseases should be considered in screening protocols.

The aim of this study was to evaluate the effect of maternal and neonatal factors on neonatal TSH levels and offer practical recommendations to improve current screening algorithms for primary CH.

## Materials and Methods

### Study Population and Screening Protocol

We retrospectively analyzed data of all babies born in Abruzzo between January 1, 2011 and December 31, 2016 and screened for primary CH by DBS TSH. The Abruzzo CH screening program, which began in 1994, is housed in Chieti at the Center of Sciences on Aging and Translational Medicine (CeSI-MeT). In most cases, blood was collected by heel prick between 48 and 120 h of life, spotted onto collection cards known as Guthrie cards (Whatman 903, Expertmed SRL, Verona, Italy), and then mailed to the CeSI-MeT laboratory. When newborns were transferred to another hospital, however, blood samples could also be collected before 48 h. Cards of poor quality and/or containing insufficient blood were considered “*inadequate*” for the assay and prompted the request of a new sample.

Cards originated from 12 hospitals (Atri, Avezzano, Chieti, L'Aquila, Lanciano, Ortona, Penne, Pescara, Sant'Omero, Sulmona, Teramo, and Vasto) located in the four provinces (Chieti, Pescara, Teramo, L'Aquila) of Abruzzo, a region that is still considered mildly iodine deficient (7). The following information was extracted from the collection cards: sex, date of birth, province of birth, age at blood collection, prematurity (reported as gestational age <37 weeks or not), birthweight, dopamine, total parenteral nutrition, blood transfusions, malformations, twin delivery, pre-gestational history of thyroid disease, and/or use of anti-thyroid drugs during pregnancy. Unfortunately, gestational age on collection cards was reported as a categoric variable (i.e., gestational age <37 or not), limiting the possibility to define the newborn as small or appropriate for gestational age. Furthermore, cards did not include information about mode of delivery, APGAR score, hematocrit, administration of glucocorticoids, and maternal iodine status.

TSH was measured using an automated time-resolved Fluoro-Immuno-Assay that uses a monoclonal antibody directed against the β subunit of human TSH (AutoDELFIA hTSH, Perkin Elmer, Waltham, MA). According to the manufacturer, the analytical sensitivity is 2 mU/L, although values comprised between 0.5 and 2 mU/L were reported by the analyzer. The screening program used a cutoff value of 7 mU/L to distinguish normal from elevated TSH, a value approximating the 98th percentile of the TSH distribution observed in term and normal weight neonates who do not have congenital hypothyroidism.

Neonates with DBS TSH values <7 mU/L were considered negative for primary CH and underwent no further testing. Those with TSH ≥ 7 mU/L were recalled for a second DBS collection; if TSH elevation was confirmed, they were considered highly suspicious for primary CH and referred to pediatrician for confirmatory testing. Neonates with TSH <7 mU/L in the initial sample but belonged to “at risk” categories (preterm, low birthweight, twins, malformations, recipients of blood transfusions, dopamine, total parenteral nutrition, and/or born to mothers with a history of autoimmune thyroid disease) underwent a routine second TSH screening after 15 days of age.

### Study Outcomes and Statistical Analysis

DBS TSH was the main outcome variable of the study and was related to the following 13 covariates: sex (male or female), calendar year of birth (2011, 2012, 2013, 2014, 2015, or 2016), season of birth (winter, spring, summer, or fall), province of birth (Chieti, Pescara, Teramo, L'Aquila), age at blood drawing (in days), use of dopamine (yes or no), total parenteral nutrition (yes or no), blood transfusions (yes or no), malformations (yes or no), maternal history of autoimmune thyroid disease (yes or no), twin delivery (yes or no), prematurity (yes or no), and birthweight (normal birthweight, NBW, 2,500–4,500 g, or abnormal birthweights). Prematurity was defined as pregnancy duration <37 weeks.

Abnormal birthweights were classified according to the four World Health Organization categories (8): high birth weight, (HBW, >4,500 g), low birth weight (LBW, 2,500–1,500 g), very low birth weight (VLBW, 1,000–1,499 g), and extremely low birth weight (ELBW, <1,000 g).

De-identified data were entered into a FileMaker database (FileMaker Pro Advanced 14.0.1, Inc., Santa Clara, CA, USA) and then analyzed using the statistical software Stata (Stata 15.1, College Station, TX, USA). We excluded from the analysis of factors influencing TSH levels, blood samples that were inadequate for the TSH assay, samples not accompanied by information about age at collection and/or birthweight, samples coming from babies born outside of the Abruzzo region, samples collected beyond 15 days of age, and samples that were classified as “highly suspicious for primary CH.”

Data were described using mean and standard deviation for normally distributed quantitative variables, median, and interquartile range for non-normally distributed quantitative variables, and frequencies and percentages for qualitative variables. Monthly birth rate was calculated by dividing the number of births in each month by the total Abruzzo population in the corresponding year, this latter information obtained from the Italian National Institute of Statistics.

The neonatal TSH percentile charts were created by calculating in healthy neonates the 10, 25, 50, 75, 90, 95, 97.5, and 99th percentiles of TSH according to sex and neonatal age. Arithmetic TSH values were transformed to a natural logarithm scale to approximate the normal distribution.

We initially performed a series of simple linear regressions where the log-transformed TSH was related individually to the 13 covariates. We then used multiple linear regression to model the log-transformed TSH based on the combination of all covariates.

In this model, prematurity, total parenteral nutrition, dopamine administration, and blood transfusion were combined into one regressor called infant factors; whereas history of maternal autoimmune thyroid disease and twin-delivery into a regressor called maternal factors. The final model, therefore, included eight covariates: sex, calendar year of birth, season of birth, province of birth, age at blood drawing, birthweight, infant factors, and maternal factors. A ninth covariate was created by multiplying prematurity by birthweight to assess their interaction.

Normal plot of residuals was used to check the normality of the residual distribution. Linearity and equal variance (homoscedasticity) of residuals were checked by examining the plot of standardized residuals. Co-linearity between predictors was tested using the test of variance inflation factor.

## Results

### Outcomes of the CH Screening

**Figure 1:**
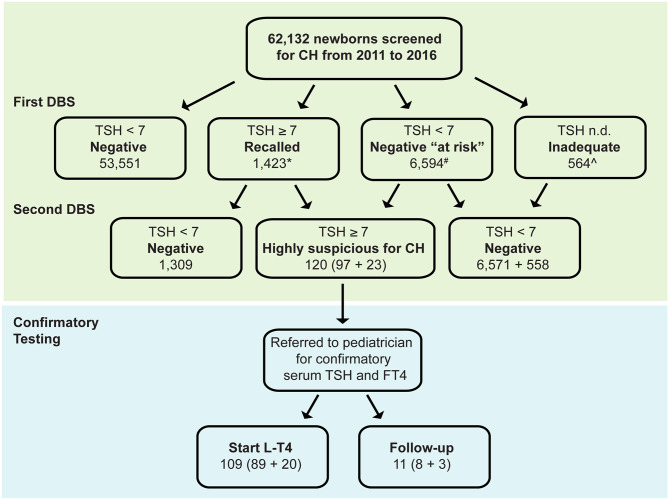
Distribution of TSH results at the initial (first dried blood spot, DBS) and subsequent testing in 62,132 babies born in Abruzzo (Italy) between January 1, 2011 and December 31, 2016. DBS, dried blood spot, *17 lost to follow-up, ^∧^6 lost to follow-up, ^#^Neonates belonging to the following categories: preterm, low birthweight, twins, malformations, blood transfusions, dopamine, total parenteral nutrition, maternal thyroid disease CH Congenital hypothyroidism, L-T4 Levothyroxine.

Between January 1, 2011 and December 31, 2016, a total of 71,743 collection cards were received at the Abruzzo regional screening center for primary CH, corresponding to 62,132 newborns. Of them, 53,551 newborns (86.2%) had a TSH value <7 mU/L and were therefore considered negative for primary CH (Figure 1).

In 1,423 newborns TSH was ≥7 mU/L in the initial screening, prompting the request of a second DBS sample, thus, yielding a recall rate of 2.3%. The second TSH measurement was abnormally elevated in 97 of 1,423 babies (7%, Figure 1) who were therefore referred to a pediatric endocrinologist for confirmatory testing (“highly suspicious for primary CH”).

In 6,594 newborns (10.6%) the TSH, although normal in the initial screening, underwent a second test because of clinical features that made them at higher risk of having a missed diagnosis of primary CH (*negative* “*at risk*,” Figure 1). Of them, 23 (0.4%) have elevated TSH on second screen and were referred to a pediatric endocrinologist for further investigation.

Of the referred 120 (97 + 23) newborns, 109 (89 + 20) had abnormal serum T4 and TSH values on confirmatory laboratory tests and initiated levothyroxine (L-T4) replacement therapy, whereas 11 (8 + 3) had normal serum T4 and normal or slightly elevated TSH, thus were classified as having “transient TSH elevation” and underwent periodic follow-up (Figure 1). The first group of newborns could not be distinguished from the latter group based on the 13 covariates analyzed in the study.

A few newborns (564 of 62,132, 0.9%) had cards of poor quality and/or containing insufficient blood (“*inadequate*”), which prompted the drawing of a second blood sample. At the second testing, the majority of them (98.9%) had normal TSH values and were considered negative for primary CH.

Overall, the period prevalence of primary CH in this 6-year interval was 0.17% (109 of 62,132, Figure 1). This value corresponded to an incidence of primary CH of 1 case every 570 births. The second screening performed in the “negative at risk” group identified three additional cases for 10,000 births. No clinical data were available to distinguish transient from permanent primary CH.

### General Characteristics of the Study Population

**Figure 2:**
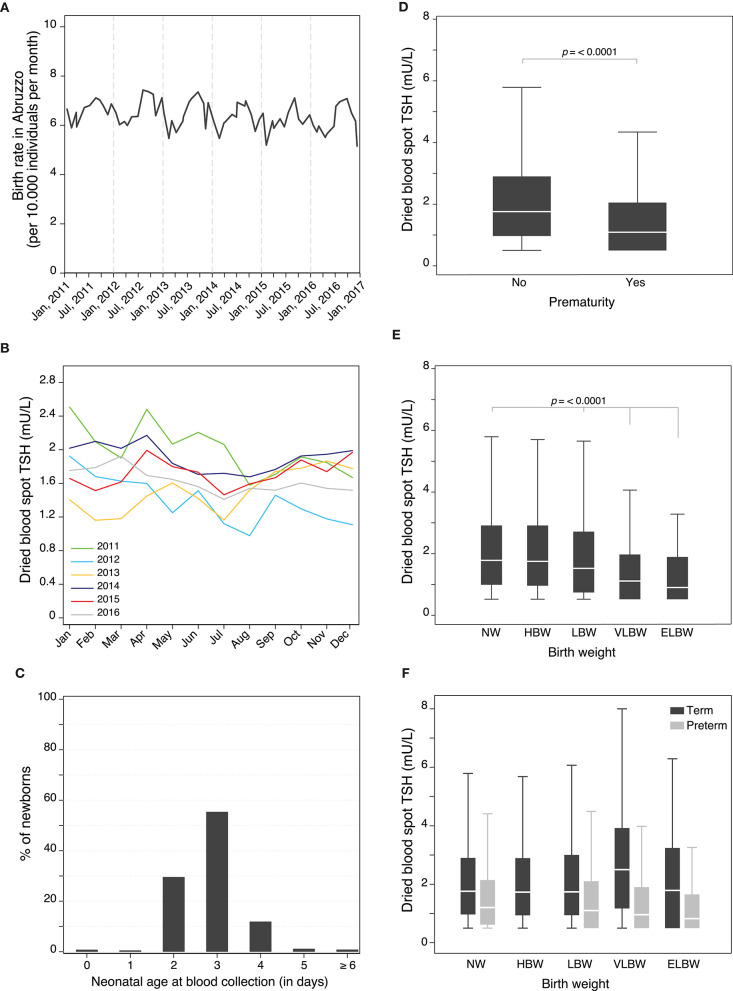
**(A)** Monthly birth rate in Abruzzo (Italy) between January 1, 2011 and December 31, 2016. **(B)** Seasonal changes in neonatal TSH during the 6-year study period. **(C)** Distribution of newborns according to their neonatal age (in days) at blood collection. Individual influence of prematurity **(D)**, and birth weight **(E)** on neonatal TSH levels. Influence of birth weight on neonatal TSH levels adjusted for prematurity **(F)**.

Of the total 62,132 screened newborns, 1,315 (2.1%) were excluded from further analysis of factors influencing TSH levels because of a lack of information about sex (6, 0.01%) or birthweight (569, 0.9%), had an age > 15 days at the first blood drawing (35, 0.06%), were born outside Abruzzo (21, 0.03%), or lacked TSH value because the collection card was unsuitable for the assay (564, 0.9%). In addition, we also removed from the further analysis of factors influencing TSH levels the 120 babies that were confirmed to have elevated TSH, and thus classified as “highly suspicious for primary CH,” to avoid the skewness that would derive from the elevated TSH values that are found in babies with thyroid dysgenesis or dyshormonogenesis (Supplementary Figure 1). The remaining study population included 60,817 newborns (Supplementary Table 1). Male newborns (31,753, 52.2%) were slightly more numerous than females (29,064, 47.8%; Supplementary Table 1). Summer months recorded the highest natality (Supplementary Table 1), with peaks in August and September (Figure 2A). Neonatal TSH was highest in winter and lowest in summer months during the 6-year study period (Figure 2B). The province of Chieti had the greatest number of births (21,837 of 670,818, 36%), followed by Pescara (13,701, 23%), Teramo (11,344, 19%), and L'Aquila (13,935, 23%) (Supplementary Table 1).

The median age at blood collection was 3 days (IQR 1), with the vast majority (98%) of newborns tested between the 2nd and 4th day of age (Figure 2C). The median body weight at birth was 3,280 g (IQR 610, Supplementary Table 1).

About 4% of the newborns were preterm, 0.2% treated with dopamine, 0.3% received total parenteral nutrition, 0.4% received blood transfusions, 0.3% had malformations, 1.2% were born to mothers with autoimmune thyroid disease, and 3.2% were twins (Supplementary Table 1).

### Individual Influence of the Risk Factors on Neonatal TSH Levels

When the 13 covariates were analyzed individually by simple linear regression for their influence on neonatal TSH (Supplementary Table 2, Figure 2, Supplementary Figure 2), the most notable effects were seen with prematurity, birthweight (Figure 2), sex, neonatal age at blood collection, blood transfusions, dopamine administration, and total parenteral nutrition (Supplementary Figure 2). Prematurity was associated with significantly lower TSH levels, with an average value of 1.1 mU/L as compared to 1.8 mU/L in term babies (*p* < 0.0001, Figure 2D). Birthweight strongly influenced the levels of TSH, which decreased in a dose-dependent fashion from high to extremely low birthweight (Figure 2E). The effect of birthweight on TSH, however, was modified by prematurity. In particular, term infants with VLBW had higher TSH values than those with NW, whereas preterm with LBW or VLBW had lower TSH values than preterm with a normal birthweight (Figure 2F). Sex and neonatal age at sample collection also markedly affected TSH levels, which declined from a median of 3.5 mU/L in males and 2 mU/L in females on day 0–1.3 mU/L in both genders on day 4, 5, and 6 (Supplementary Figure 1A). Blood transfusions (Supplementary Figure 2B), dopamine administration (Supplementary Figure 2C), and total parenteral nutrition (Supplementary Figure 2D) were associated with significantly lower TSH levels. Interestingly, seasons significantly affected TSH levels, with the peak value observed in winter (Supplementary Figure 3 and Supplementary Table 2). The individual effect of the remaining covariates was less pronounced, although reaching statistical significance (summarized in Supplementary Figure 3 and Supplementary Table 2).

### Combined Influence of the Risk Factors on Neonatal TSH Levels

**Figure 3:**
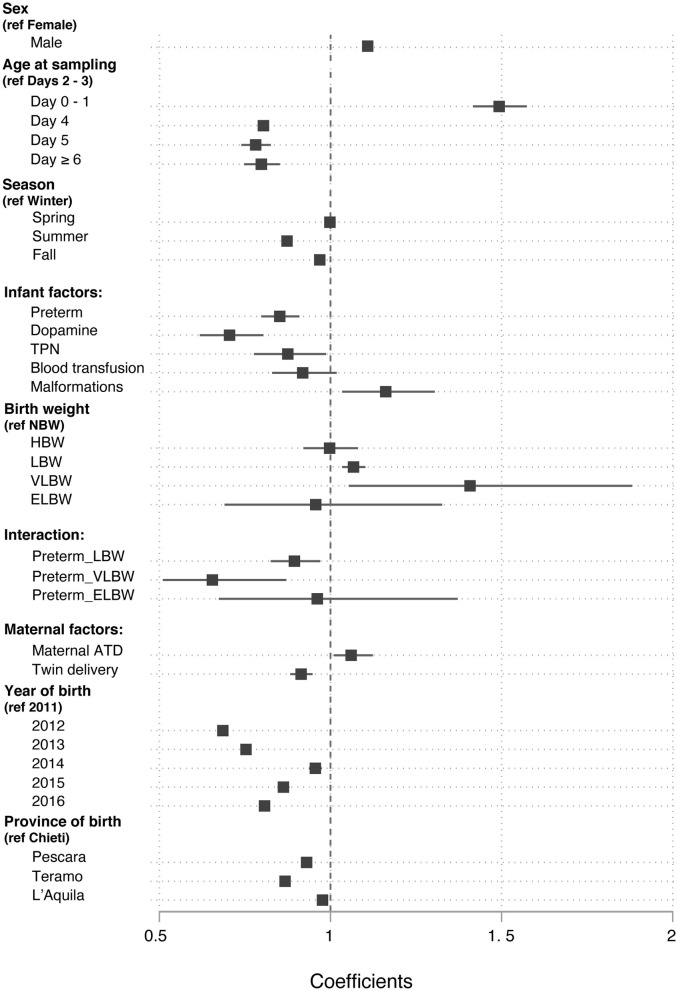
Values of the β regression coefficients in a multiple linear regression model that related the neonatal blood TSH levels to a set of nine covariates.

When the covariates were analyzed in a multiple linear regression model that related them to DBS TSH, most of them retained statistical significance after adjusting for the influence of the others (Table 1 and Figure 3). The strength of their association was modest, with regression coefficients ranging from a 50% decrease to 40% increase in TSH levels, none of them causing a 2-fold or greater effect (Figure 3).

**Table 1:** Multiple linear regression analysis of the factors affecting neonatal TSH.

	**ß**	**Standard error**	**(95% Confidence Interval)**		***p*-value**
ß_0_	3.54	0.12	3.31	2.3.80	<0.0001
Sex, Male (Female ref)	1.11	0.01	1.09	1.12	<0.0001
**Neonatal age at blood collection (Day 2–3 ref)**					
Day <2	1.49	0.04	1.42	1.57	<0.0001
Day 4	0.80	0.01	0.79	0.82	<0.0001
Day 5	0.78	0.02	0.74	0.83	<0.0001
Day ≥ 6	0.80	0.03	0.75	0.85	<0.0001
**Season of birth (Winter ref)**					
Spring	1.00	0.01	0.98	1.01	0.817
Summer	0.87	0.01	0.86	0.89	<0.0001
Fall	0.98	0.01	0.95	0.98	<0.0001
**Infant factors**					
Preterm	0.85	0.03	0.80	0.91	<0.0001
Dopamine	0.71	0.05	0.62	0.81	<0.0001
Total parental nutrition	0.88	0.05	0.78	0.99	0.034
Blood transfusions	0.92	0.05	0.83	1.01	0.110
Malformations	1.17	0.07	1.04	1.31	0.010
**Birth Weight (ref NW)**					
HBW	1.00	0.04	0.93	1.10	0.947
LBW	1.08	0.02	1.04	1.11	<0.0001
VLBW	1.40	0.21	1.05	1.89	0.023
ELBW	0.99	0.16	0.70	1.34	0.853
**Interaction term**					
LBW × Preterm	0.89	0.04	0.82	0.96	0.004
VLBW × Preterm	0.65	0.10	0.48	0.89	0.023
ELBW × Preterm	0.97	0.16	0.69	1.34	0.853
**Maternal factors**					
Maternal autoimmune TD	1.10	0.03	1.01	1.13	0.016
Twin delivery	0.92	0.02	0.89	0.96	<0.0001
**Year of birth (2011 ref)**					
2012	0.68	0.01	0.67	0.70	<0.0001
2013	0.75	0.01	0.74	0.77	<0.0001
2014	0.95	0.01	0.93	0.97	<0.0001
2015	0.86	0.01	0.84	0.88	<0.0001
2016	0.80	0.01	0.79	0.82	<0.0001
**Province of birth (Chieti ref)**					
Pescara	0.92	0.01	0.91	0.94	<0.0001
Teramo	0.85	0.01	0.84	0.87	<0.0001
L'Aquila	0.96	0.01	0.94	0.97	<0.0001
Observations 60,817 *R*^2^ 0.36					

#### Infant Factors

Neonatal TSH was influenced by sex: males had higher TSH levels than females (β regression coefficient, β = 1.11; 95% CI 1.09, 1.12; *p* < 0.0001). Neonatal TSH, as expected, was higher on day 0 and 1 compared to days 2–3 (β = 1.49; 95% CI 1.42, 1.57; *p* < 0.0001), whereas it showed a negative correlation with age when measured on day 4, 5, or 6 of life than on days 2–3 (β = 0.78; 95% CI 0.74, 0.83; *p* < 0.0001).

The season of birth also influenced the neonatal TSH levels. Neonates born in summer and autumn had a 13% (β = 0.87; 95% CI 0.86, 0.89; *p* < 0.0001) and 2% (β = 0.98; 95% CI 0.95, 0.98; *p* < 0.0001) lower TSH than those born in winter, respectively.

Preterm had a 15% lower TSH than term infants (β = 0.85; 95% CI 0.80, 0.91; *p* < 0.0001). Moreover, the use of dopamine (β = 0.71; 95% CI 0.62, 0.81; *p* < 0.0001), total parenteral nutrition (β = 0.88; 95% CI 0.78, 0.99; *p* = 0.034), and malformations (β = 1.17; 95% CI 1.04, 1.34; *p* = 0.01) were also significantly associated with TSH. Administration of blood transfusion, on the contrary, had no significant effect on neonatal TSH.

In the univariate analysis, TSH was lower in LBW, VLBW, and ELBW than in NW newborns. However, in the final multiple linear regression model TSH was higher in LBW (β = 1.08; 95% CI 1.04, 1.11; *p* < 0.0001) and in VLBW (β = 1.40; 95% CI 1.05, 1.89; *p* = 0.023) compared to NW newborns, after adjusting for prematurity. The relationship between birthweight and TSH was also influenced by gestational age. Indeed, preterm newborns with LBW (β = 0.89; 95% CI 0.82, 0.96; *p* = 0.004) and VLBW (β = 0.65; 95% CI 0.48, 0.89; *p* = 0.023) had lower TSH levels than term infants.

#### Maternal Factors

Maternal history of pre-gestational autoimmune thyroid disease was associated with higher neonatal TSH levels in the multiple linear regression analysis (β = 1.10; 95% CI 1.01, 1.13; *p* = 0.016). Twins had an 8% lower TSH value than singletons (β = 0.92; 95% CI 0.89, 0.96; *p* < 0.0001).

### Calculation of Neonatal TSH Percentile Charts Based on Sex and Age

**Figure 4:**
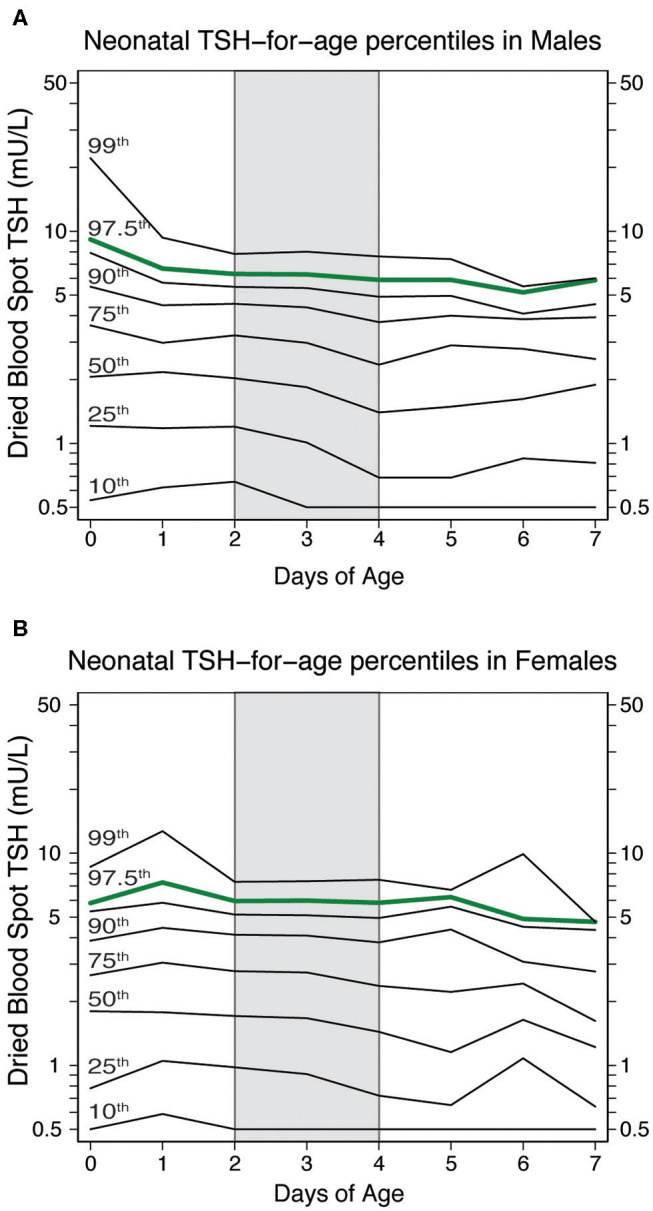
Neonatal TSH percentiles charts in healthy neonates according to their neonatal age and sex [(males in panel **(A)** and females in panel **(B)**]. The green line represents the 97.5th percentiles of neonatal TSH, which could be used to establish TSH cut-off value at the screening centers.

The significant effect of sex and age at blood collection had on neonatal TSH levels prompted us to devise percentile charts illustrating the distribution of TSH values in healthy neonates (i.e., at term, normo-weight, not affected by primary CH, Figure 4). The charts include eight lines corresponding to the 99, 97.5, 95, 90, 75, 50, 25 and 10th percentile. They depict the slightly elevated TSH values in males during the first two days of life and a more stable trend in the upper quartile of the distribution between the 2nd and 4th day of life (shaded area in Figure 4). Local percentile charts may be a useful tool to identify elevated TSH values at each day of postnatal age in each sex.

## Discussion

We utilized newborn screening data over a 6-year period to examine the associations between TSH levels and several infant and maternal factors. This study revealed that neonatal TSH is influenced by several factors including sex, age at blood collection, prematurity, use of dopamine and total parenteral nutrition, birthweight, season of birth, history of autoimmune maternal thyroid disease, and twin-pregnancy.

Males had significantly higher TSH levels than females in our study population, in keeping with what has been reported by most studies (9–13). Some studies, however, have failed to show this sex difference (14–17), likely due to smaller sample sizes and/or a focus on preterm newborns. The mechanisms underlying the elevated neonatal TSH levels in males remains unexplained. From a practical perspective, however, this difference should encourage primary CH screening programs to devise their own sex-specific cut-off values. When coupled with age-specific cut-offs, local percentile charts, as we have developed in this study, could improve the accuracy of primary CH screening. The TSH for age and sex percentile charts also underscore the importance of timing the blood collection between the second and fourth day of life (4), so as not to miss cases where TSH is mildly but persistently elevated (18).

The effect of prematurity on neonatal TSH has been extensively investigated. Some studies found lower TSH levels in preterm newborns than in those born after 37 weeks of gestation (10, 19, 20), which is also what we found. Other studies, instead, reported higher TSH values in preterm infants (11, 12, 17), and others no difference in TSH levels according to gestational age (9, 15). Since it has been shown that preterm babies have an immature hypothalamo-pituitary axis (21), it is reasonable to postulate that preterm infants indeed have lower TSH levels and ascribe the variability to differences in blood collection timing and study populations. It has also been proposed that other conditions occurring in preterm, such as drug exposure [dopamine (22), glucocorticoids (23)], total parenteral nutrition (24), sepsis (25), and respiratory distress syndrome (25) decrease TSH values in preterm babies. In our study, the administration of dopamine and total parenteral nutrition was indeed associated with lower TSH levels after controlling for preterm status. However, the lower TSH levels observed in newborns treated with dopamine and TPN may be related to concomitant severe illness, not considered in our multivariate analysis.

The influence of birthweight on TSH remains debatable. Several studies (10–12, 17, 26) have reported higher TSH levels in LBW and VLBW babies than in NW babies. When the effect was analyzed in a multiple regression model, however, we noted it was modified by prematurity. In particular, preterm babies with LBW or VLBW had lower TSH values than those with a normal birthweight, whereas term infants with VLBW had higher TSH values than those with NW. Our findings suggest that preterm infants with LBW or VLBW require repeated TSH testing to properly establish a diagnosis of primary CH, as highly recommended by Hashemipour et al. (6) who systematically reviewed previous works on screening for CH in preterm, LBW, and VLBW infants.

The influence of the season of birth on neonatal TSH has been reported in Belgium (16, 26), Latvia (27), Turkey (28), Iran (29, 30), and Iowa (19), but never in Italy. We found that neonatal TSH is significantly higher in winter than summer and fall, in keeping with what has been reported in Turkey, Iran and Iowa. In Belgium and Latvia, on the contrary, TSH levels were found to be higher during the fall months. The cause(s) of this seasonal variation is not known. A decreased maternal consumption of iodine-rich foods during the winter months could lead to increased TSH levels in the baby. Alternatively, lower environmental temperatures could stimulate the fetal pituitary to produce more TSH. In adults, TSH hypersecretion secondary to low ambient temperatures has indeed been reported in patients with primary hypothyroidism on constant replacement dosage of thyroxine (31). Recently, Yoshihara et al. (32) reported seasonal changes in TSH in 135,417 Japanese adult patients and a negative correlation between TSH and daily temperature. Another possible explanation is the relationship between maternal vitamin D and fetal TSH levels. Barchetta et al. (33) and Das et al. (34) have reported that TSH is higher in winter and inversely correlated with vitamin D levels in euthyroid adults, offering a mechanism to explain TSH seasonality.

Although the history of maternal thyroid disease is generally considered a risk factor for CH (35–37), few studies have actually examined its effect on neonatal TSH. Some authors (15, 16, 25) did not observe a relationship between history of maternal thyroid dysfunction and/or thyroid nodules, and neonatal TSH, either in univariate models or after adjusting for gestational age. Others, focusing on the relation between autoimmune thyroid disease and neonatal TSH levels, reported higher TSH values in neonates born to mothers with autoimmune thyroid disease (38–40). Consistent with the latter studies, we found that newborns born to mothers with pre-gestational history of autoimmune thyroid disease had higher TSH levels in multivariable, but not univariate, models. The trans-placental passage of antibodies blocking the TSH receptor and/or the use of anti-thyroid drugs could be responsible for these neonatal thyroid dysfunctions. However, we did not specifically evaluate the influence of anti-thyroid drugs on neonatal TSH levels.

Twin deliveries represent a challenge for primary CH screening. Our twins had lower TSH than singletons, in keeping with the hypothesis that twins have a reduced post-natal rise in TSH likely due to mixing of fetal blood in monozygotic twins and increased risk of preterm delivery. We consider twins at greater risk of missed or delayed primary CH diagnosis (41–44), and confirm the use of special TSH screening protocols for this category of newborns. The effect of twin pregnancy on TSH, however, is not invariably reported. Ryckman et al. (25) and Bosch-Gimenez et al. (14) reported no effect of twin pregnancy on TSH levels in preterm newborns. In addition, Lee (15) showed that the first of twin babies had higher TSH levels than singletons in univariate analysis. In their multiple regression model, only birth order influenced TSH levels, suggesting that a greater stress occurring during delivery, rather than the fact of being a twin, modified neonatal TSH levels.

Strengths of our study are the comprehensive assessment of the main infant and maternal factors that have been reported to influence neonatal TSH, as well as the large size and unbiased nature of the study population. The multivariate analysis performed is another strength, as it provides the true influence on neonatal TSH of the variables analyzed. Weaknesses are related mainly to the quality of the data source, that is the information recorded on the collection cards. For example, cards listed the gestational age as term or prematurity, rather than a true number, a simplification that limited the possibility to define a neonate small or appropriate for gestational age and thus to better clarify the interaction between birthweight and prematurity. In addition, our collection cards did not include other factors known to influence neonatal TSH [such as, maternal origin (14), maternal thyroid function during pregnancy (25), iodine supplementation (16), mode of delivery (15), APGAR score (45), and hematocrit (46)], highlighting the need of replicating our findings in more extended datasets. One major limitation is that we lacked measures of iodine status. The Abruzzo region can be considered a mildly iodine deficient area (47). Therefore, we cannot exclude that iodine deficiency, even if mild, may have contributed for any alteration in measured TSH levels in this population.

In conclusion, this study highlights the influence of several infant and maternal factors on neonatal TSH supporting the delineation of own sex- and age-specific TSH cutoff values that can be used to refine local screening protocols. The study also supports the universal adoption of a second TSH screening in neonates at risk of missed primary CH detection.

## Data Availability Statement

The datasets generated for this study are available on request to the corresponding author.

## Ethics Statement

Ethical review and approval was not required for the study on human participants in accordance with the local legislation and institutional requirements. Written informed consent for participation was not required for this study in accordance with the national legislation and the institutional requirements.

## Author Contributions

GD contributed to the design/plan of the study, performed the statistical analysis, wrote, and revised the manuscript. MC contributed to data collection and revision of the manuscript. DS performed the TSH measurements and contributed to data collection and revision of the manuscript. CG contributed to the study design and revision of the manuscript. GN directs the neonatal screening center and contributed to the study design and revision of the manuscript. PC contributed to the writing and revision of the manuscript. IB co-directs the neonatal screening and contributed to the writing and revision of the manuscript. All authors contributed to the article and approved the submitted version.

## Conflict of Interest

The authors declare that the research was conducted in the absence of any commercial or financial relationships that could be construed as a potential conflict of interest.
