# Supplementary figures and images for: A Detailed Analysis of the Factors Influencing Neonatal TSH: Results From a 6-Year Congenital Hypothyroidism Screening Program
Source: Front Endocrinol (Lausanne). 2020 Jul 17;11:456. doi: 10.3389/fendo.2020.00456 (PMC7396660; doi:10.3389/fendo.2020.00456)

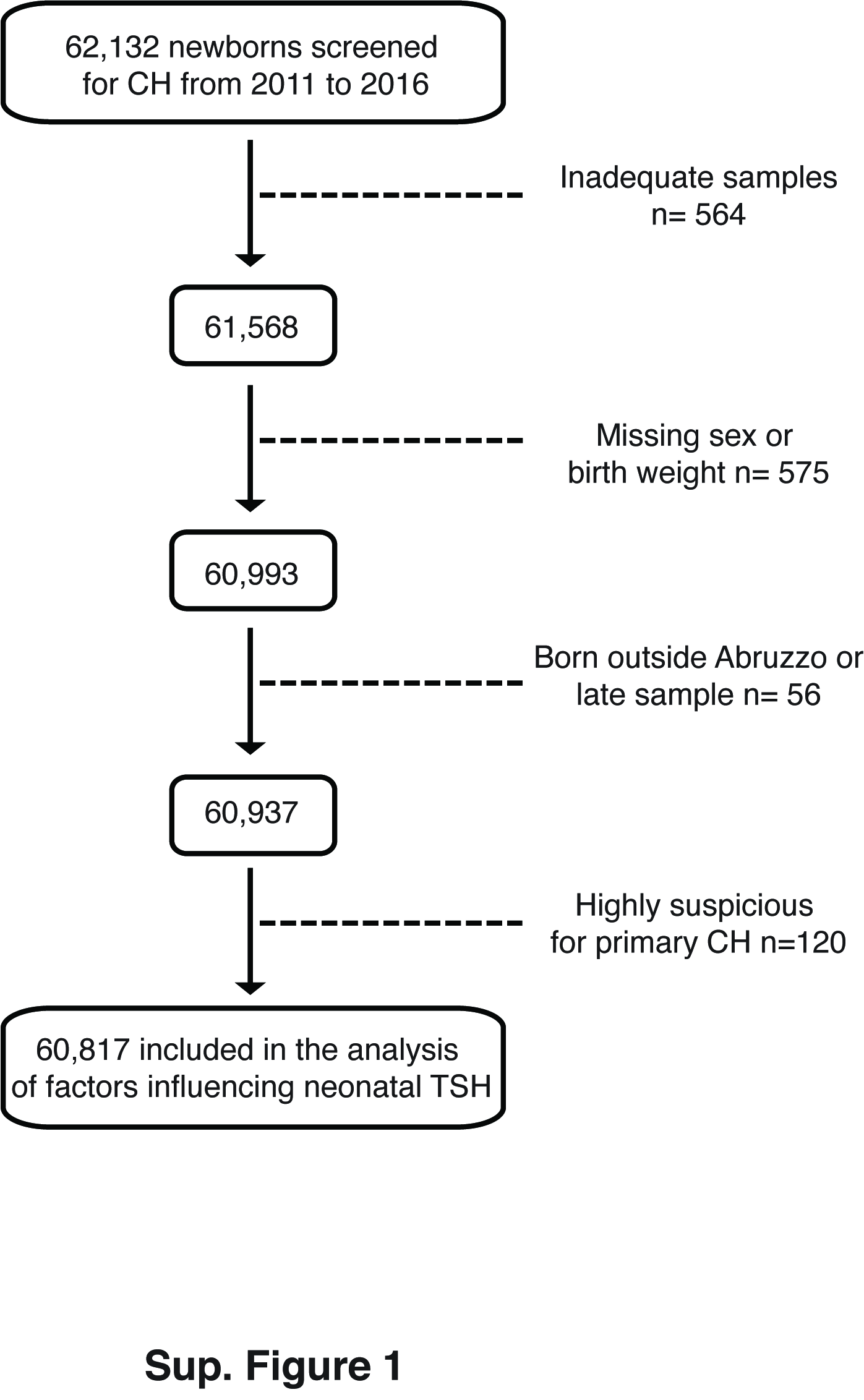

Supplement: Supplementary file 1 [file Image_1.TIF]

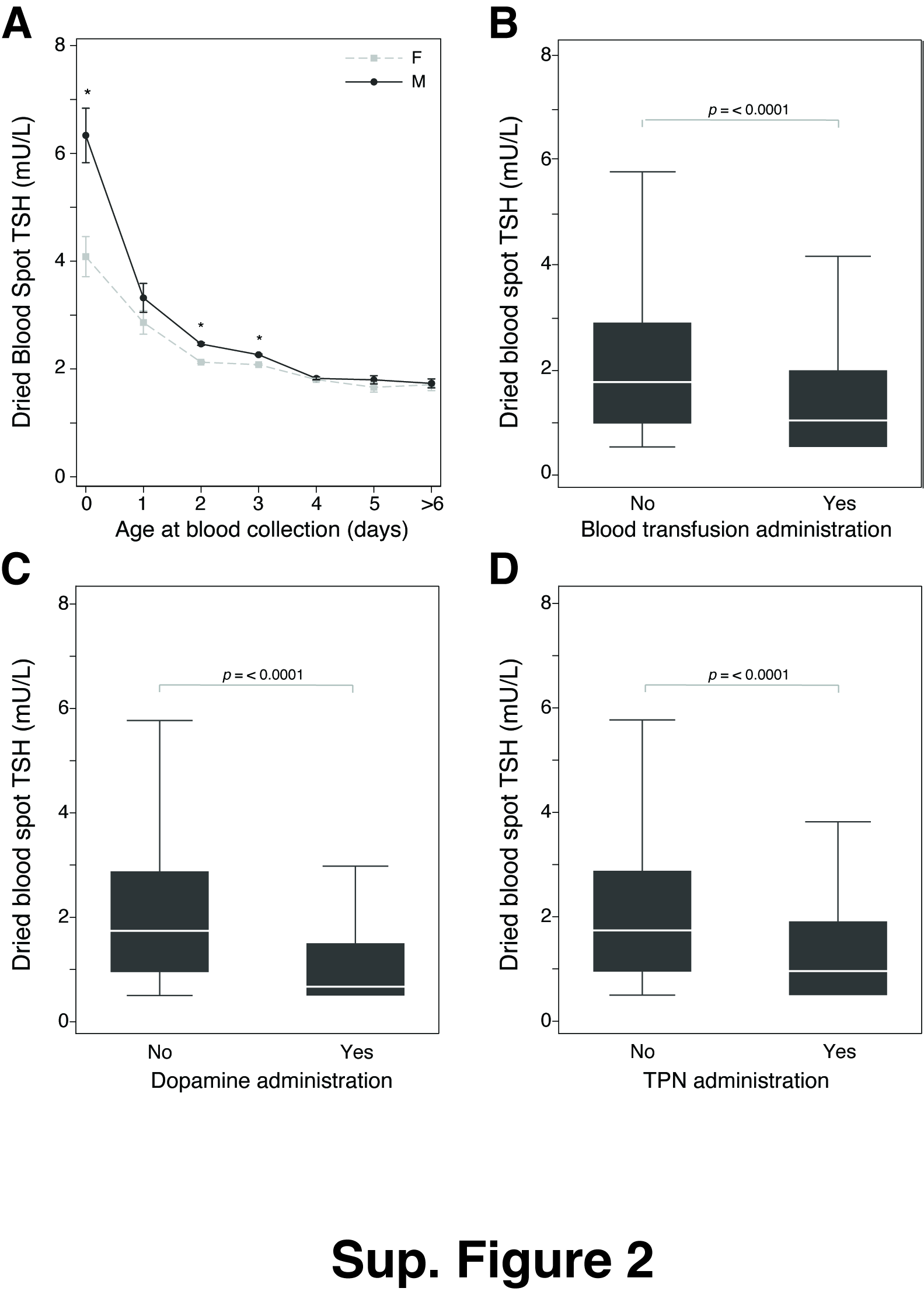

Supplement: Supplementary file 2 [file Image_2.TIF]

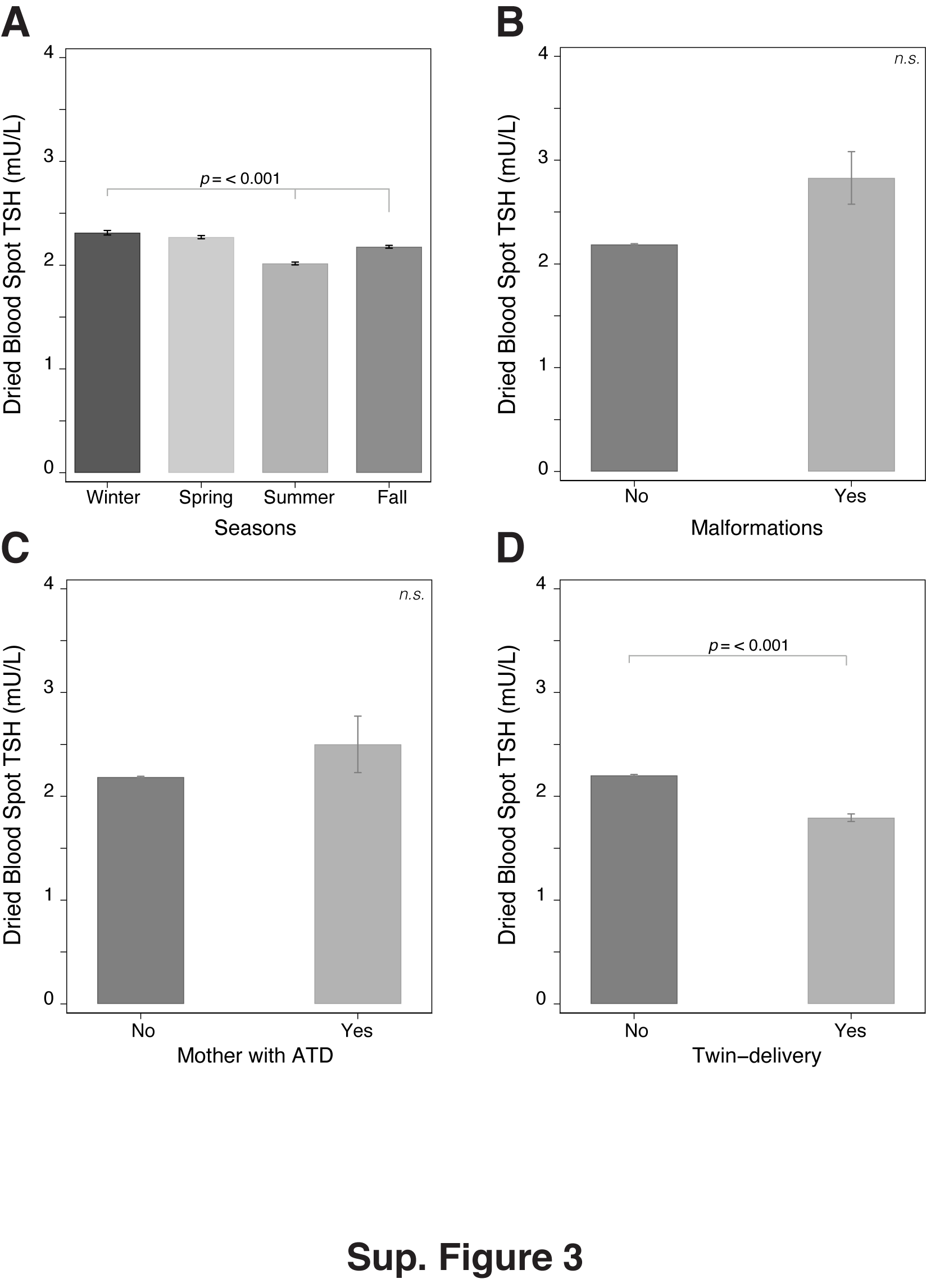

Supplement: Supplementary file 3 [file Image_3.TIF]
