# Supplementary material for: A Detailed Analysis of the Factors Influencing Neonatal TSH: Results From a 6-Year Congenital Hypothyroidism Screening Program
Source: Front Endocrinol (Lausanne). 2020 Jul 17;11:456. doi: 10.3389/fendo.2020.00456 (PMC7396660; doi:10.3389/fendo.2020.00456)
Supplement: Supplementary file 4 [file Table_1.DOCX]

**Supplemental Table 1 (eTable1)**

General characteristics of the study population: 60,817 infants born in Abruzzo (Italy) between 2011 and 2016 screened for congenital hypothyroidism.

| **Year of birth** | **2011** | **2012** | **2013** | **2014** | **2015** | **2016** | **Totals** |
| --- | --- | --- | --- | --- | --- | --- | --- |
| No. and sex of newborns: | 10,403 | 10,467 | 10,169 | 10,107 | 9,926 | 9,746 | 60,817 |
| males | 5,425 | 5,420 | 5,288 | 5,279 | 5,165 | 5,176 | 31,753 |
| females | 4,978 | 5,047 | 4,881 | 4,828 | 4,761 | 4,569 | 29,064 |
| No. born in:  winter  spring  summer  fall | 2,488  2,527  2,730  2,685 | 2,497  2,451  2.792  2,727 | 2,395  2,428  2,734  2,612 | 2,325  2,515  2,744  2,523 | 2,415  2,437  2,549  2,524 | 2,245  2,297  2,727  2,477 | 14,365  14,656  16,276  15,521 |
| No. born in the province of:  Chieti  Pescara  Teramo  L’Aquila | 3,746  2,413  1,861  2,383 | 3,671  2,333  2,021  2,442 | 3,443  2,406  2,024  2,296 | 3,633  2,206  1,999  2,269 | 3,703  2,031  1,855  2,337 | 3,641  2,312  1,584  2,208 | 21,837  13,701  11,344  13,935 |
| Median (IQR) age at blood collection (days) | 3 (1) | 3 (1) | 3 (1) | 3 (1) | 3 (1) | 3 (1) | 3 (1) |
| Median (IQR) body weight at birth (g) | 3,270  (620) | 3,290  (600) | 3,280  (600) | 3,280  (610) | 3,260  (605) | 3,275  (600) | 3,280  (610) |
| No. (%) of newborns with risk factors: |  |  |  |  |  |  |  |
| Preterm | 166  (1.6) | 434  (4.2) | 403  (3.9) | 422  (4.2) | 474  (4.8) | 396  (4.1) | 2,295  (3.8) |
| Dopamine | 25  (0.2) | 26  (0.3) | 15  (0.2) | 26  (0.3) | 15  (0.2) | 14  (0.1) | 121  (0.2) |
| Total parenteral nutrition | 2  (0.02) | 7  (0.1) | 38  (0.4) | 43  (0.4) | 25  (0.3) | 36  (0.4) | 151  (0.3) |
| Blood transfusions | 32  (0.3) | 34  (0.3) | 45  (0.4) | 57  (0.6) | 27  (0.3) | 33  (0.3) | 228  (0.4) |
| Malformations | 18  (0.2) | 23  (0.2) | 25  (0.3) | 30  (0.3) | 35  (0.4) | 19  (0.2) | 150  (0.3) |
| Mother with ATD | 91  (0.9) | 111 (1.1) | 128 (1.3) | 143 (1.4) | 127 (1.3) | 114 (1.2) | 714 (1.2) |
| Twins | 346  (3.3) | 339  (3.2) | 332  (3.3) | 291  (2.9) | 311  (3.1) | 321  (3.3) | 1,940  (3.2) |
