# Supplementary material for: A Detailed Analysis of the Factors Influencing Neonatal TSH: Results From a 6-Year Congenital Hypothyroidism Screening Program
Source: Front Endocrinol (Lausanne). 2020 Jul 17;11:456. doi: 10.3389/fendo.2020.00456 (PMC7396660; doi:10.3389/fendo.2020.00456)
Supplement: Supplementary file 5 [file Table_2.docx]

| **Supplemental Table 2 (eTable2).**  Simple linear regression analysis of the factors affecting neonatal TSH | | | | | |
| --- | --- | --- | --- | --- | --- |
| **Variable** | **ß** | **Std. Err.** | **[95% Conf. Interval]** | | ***p value*** |
| **Prematurity (No ref)**  **Yes** | -0.35 | 0.02 | -0.38 | -0.32 | 0.000 |
| **Birth weight (NW ref)** |  |  |  |  |  |
| **HW** | 0.00 | 0.04 | -0.08 | 0.08 | 0.996 |
| **LBW** | -0.12 | 0.01 | -0.15 | -0.99 | 0.000 |
| **VLBW** | -0.38 | 0.04 | -0.46 | -0.29 | 0.000 |
| **ELBW** | -0.44 | 0.06 | -0.56 | -0.32 | 0.000 |
| **Neonatal age at blood collection (Day 2-3 ref)** |  |  |  |  |  |
| **Day < 2** | 0.40 | 0.03 | 0.35 | 0.45 | 0.000 |
| **Day 4** | -0.24 | 0.01 | -0.26 | -0.23 | 0.000 |
| **Day 5** | -0.29 | 0.03 | -0.34 | -0.23 | 0.000 |
| **Day ≥ 6** | -0.27 | 0.03 | -0.33 | -0.20 | 0.000 |
| **Sex (Females ref)**  **Males** | 0.10 | 0.01 | 0.09 | 0.11 | 0.000 |
| **Blood transfusions (No ref)**  **Yes** | -0.39 | 0.05 | -0.49 | -0.29 | 0.000 |
| **Dopamine (No ref)**  **Yes** | -0.57 | 0.07 | -0.70 | -0.44 | 0.000 |
| **TPN (No ref)**  **Yes** | -0.42 | 0.06 | -0.54 | -0.30 | 0.000 |
| **Year of birth (2011 ref)** |  |  |  |  |  |
| **2012** | -0.38 | 0.01 | -0.40 | -0.36 | 0.000 |
| **2013** | -0.29 | 0.01 | -0.30 | -0.26 | 0.000 |
| **2014** | -0.04 | 0.01 | -0.06 | -0.02 | 0.000 |
| **2015** | -0.14 | 0.01 | -0.16 | -0.12 | 0.000 |
| **2016** | -0.20 | 0.01 | -0.22 | -0.18 | 0.000 |
| **Season of birth (Winter ref)** |  |  |  |  |  |
| **Spring** | 0.00 | 0.01 | -0.01 | 0.02 | 0.802 |
| **Summer** | -0.13 | 0.01 | -0.15 | -0.11 | 0.000 |
| **Fall** | -0.03 | 0.01 | -0.05 | -0.01 | 0.001 |
| **Province of birth (Chieti ref)** |  |  |  |  |  |
| **Pescara** | -0.07 | 0.01 | -0.08 | -0.05 | 0.000 |
| **Teramo** | -0.16 | 0.01 | -0.18 | -0.15 | 0.000 |
| **L’Aquila** | -0.01 | 0.01 | -0.02 | 0.00 | 0.263 |
| **Malformations (No ref)**  **Yes** | 0.07 | 0.06 | -0.05 | 0.19 | 0.286 |
| **Mother with ATD (No ref)**  **Yes** | 0.02 | 0.03 | -0.04 | 0.07 | 0.585 |
| **Twin-delivery (No ref)**  **Yes** | -0.21 | 0.02 | -0.24 | -0.18 | 0.000 |
| Observations 60,817 |  |  |  |  |  |
