# Supplementary material for: A Detailed Analysis of the Factors Influencing Neonatal TSH: Results From a 6-Year Congenital Hypothyroidism Screening Program
Source: Front Endocrinol (Lausanne). 2020 Jul 17;11:456. doi: 10.3389/fendo.2020.00456 (PMC7396660; doi:10.3389/fendo.2020.00456)
Supplement: Supplementary file 6 [file Data_Sheet_1.docx]

**Supplemental Figure Legend**

**Supplemental Figure 1.** Flow chart for eligible screening samples from newborns in Abruzzo in 2011‐2016.

**Supplemental Figure 2.** Individual influence of sex and age at blood collection (A), blood transfusions (B), dopamine (C), and total parental nutrition (D) on neonatal TSH levels. ** p<0.001*

**Supplemental Figure 3.** Individual influence of season of birth (A), malformations (B), history of maternal autoimmune thyroid disease (C), and twin-delivery (D) on neonatal TSH levels.
